# Factors influencing poor medication adherence amongst patients with chronic disease in low-and-middle-income countries: A systematic scoping review

**DOI:** 10.1016/j.heliyon.2022.e09716

**Published:** 2022-06-15

**Authors:** Gloria Dunisani Chauke, Olivia Nakwafila, Buyisile Chibi, Benn Sartorius, Tivani Mashamba-Thompson

**Affiliations:** a Discipline of Public Health Medicine, School of Nursing and Public Health, University of KwaZulu-Natal, Durban, South Africa; b Department of Public Health, School of Nursing and Public Health, University of Namibia, Oshakati Campus, Namibia; c Centres for Tropical Medicine and Global Health, Nuffield Department of Medicine, University of Oxford, Oxford, UK; d Department of Health Metric Sciences, University of Washington, Seattle, USA; e Department of Health Metrics Sciences, University of Washington, Seattle, WA, 98195, USA; f Faculty of Health Sciences, University of Pretoria, Pretoria Province, South Africa

**Keywords:** Chronic disease, Adherence and medication, Low-and middle-income countries

## Abstract

**Background:**

Poor medication adherence among patients with Chronic Diseases is one of the significant health problems globally. Despite this, evidence on chronic medication adherence in low and middle-income countries is unclear.

**Objective:**

This scoping review aimed to identify factors influencing poor medication adherence amongst patients with chronic diseases in low and middle-income countries.

**Methods:**

We searched studies exploring factors influencing poor medication adherence amongst patients in low and middle-income countries across the following databases published between 2008 to 2018: Public or publisher Medline, Google scholar, Cumulated Index to Nursing and Allied Health Literature, Health Source, and Medline with full text via Elton B. Stephen's Company host. Methodological quality assessment of the primary studies was done as recommended by Levac*,* Colquhoun, and O'Brien (2010) review using a Mixed-Method Appraisal Tool 2018. We reported the results following the Preferred Reporting Item for Systematic reviews and Meta-Analyses extension for Scoping Review guidelines.

**Results:**

From the initial 154 records screened, we identified six (6) eligible studies that presented evidence on factors influencing poor medication adherence amongst patients in low and middle-income countries. Studies included were from the following countries: Jordan, South Africa, Guatemala, Ethiopia, Indonesia, India, and Palestine. Kappa agreement of the full article screening shows that there was 76.92% agreement versus 58.12% expected by chance which constitutes a considerably good agreement between screeners (Kappa statistic = 0.45 and p-value <0.05). Of the six included studies that underwent methodological quality, five scored 100%, which is regarded as the highest score the remaining one scored between 50–75%, indicating a moderate to low risk bias overall. All included studies presented evidence on medication adherence as being in either knowledge of the diseases, attitudes towards medication taking, beliefs that a patient holds about the treatment or disease, and quality control amongst chronic diseases patients.

**Conclusions:**

Our scoping review provides evidence that poor medication adherence in LMICs is influenced by a lack of knowledge, negative attitudes, and negative beliefs, leading to poor quality of life. There is limited research evidence on the effect of patients' beliefs and perceptions on medication adherence in low and middle-income countries. We call upon further research on beliefs, perceptions, and effectiveness of interventions towards chronic medication adherence in low and middle-income countries.

## 1 Introduction

Chronic Diseases (ChDs) refer to illnesses that occur for more than six months without going away, irrespective of medication taking, leading to death or disability [1]. Chronic medication taking is often associated with poor medication adherence, which is how the patient exhibits ineffective medication management in taking a prescription, following proper diets, and executing a lifestyle change [2]. Conversely, the World Health Organization (WHO) (2003) describes adherence as the extent to which a person's behavior—taking medication, following a diet, or executing lifestyle changes—corresponds with agreed recommendations from a healthcare provider [3]. Adherence to long-term therapy for chronic diseases is estimated at 50% in high-income countries; however, in low and middle-income countries, the rates are estimated to be lower [3]. Additionally, noncommunicable diseases (NCDs) and mental disorders, human immunodeficiency virus/acquired immunodeficiency syndrome, and tuberculosis represented 54% of the burden of all diseases worldwide [3]. The consequences of poor adherence to long-term therapies is attributed to poor health outcomes and increased health care costs [3]. Adherence is simultaneously influenced by multiple factors such as social and economic factors, health care team/system, characteristics of the disease, disease therapies, and patient-related factors [3, 4]. It is necessary to tackle issues associated with each factor to improve adherence to therapy [3].

As a result of the Epidemiological transition in LMICs, WHO has developed several guidelines and models, including the package of Essential Noncommunicable diseases (PEN) and the five WHO dimensions of medication adherence [3, 5], to help guide chronic medication adherence strategies in low resource settings. Many studies focused on medication adherence related to some specific illness instead of medication adherence in general [6, 7, 8, 9, 10, 11]. Hence, it is necessary to conduct a systematic review of chronic medication adherence in general, especially in LMICs. Findings from a systematic review conducted among adult patients with chronic physical conditions identified that specific factors such as higher education and employment are attributed to good adherence. Another scoping review among patients with chronic conditions found that information and knowledge of diseases and their treatment, communication, trust in patient-provider relationships, support, and adequate resources influence medication adherence positively [12]. A Ghanian study on diabetes and hypertension reported that nonadherence resulted from perceptions that the medications are ineffective for managing these conditions [6]. Findings from the same study conducted in Ghana further outline that patients with these perceptions rejected the medications and turned to herbal medicines and spiritual healing as therapeutic alternatives because of their easy accessibility, perceived efficacy, and affordability [6]. Other factors identified to influence nonadherence included polypharmacy practice, tight work schedules; social norms; poor prescription instruction by health providers, and knowledge and experience of medication [6]. The same factors that improve medication adherence may also decrease it [3, 8, 13].

Apart from the many theories developed, there is no single theory to explain chronic medication adherence [12]. Moreso, a program such as Package of Essential Non-communicable (PEN) Disease Interventions for Primary Health Care in Low-Resource Settings have given guidance on barriers and enablers of NCDs, yet evidence of adherence to medication in patients with ChDs in the LMCs is unclear.

### 1.1 Objectives

This study aimed to map evidence of factors influencing poor medication adherence amongst patients with chronic medication in LMICs. It is anticipated that the findings of this study would assist in identifying research gaps and create a platform for future investigation into medication adherence in LMICs.

## 2 Methods

The review was guided by the Arksey and O′ Marley framework (2005), which stipulates the following five steps, namely: (1) Identification of the research question, (2) Identification of relevant studies, (3) Selection of relevant studies, (4) Data charting, (5) Result collation, summarising, and reporting. In this scoping review, we included an additional step, methodological quality assessment of the primary studies, as recommended by Levac*,* Colquhoun, and O'Brien (2010) review [14]. The protocol of this scoping review has not been registered. The results were reported following the Scoping Reviews) guidelines.

### 2.1 Eligibility criteria

This scoping review focused on mapping evidence of factors influencing medication adherence in patients with ChDs in LMICs. To determine the eligibility of the research question, we used the population, concept, and context (PCC) nomenclature. Refer to (Table 1).

**Table 1:** Eligibility of the research questions.

Population	Patients diagnosed with chronic diseases
Concept	Medication adherence
Context	Knowledge of the disease and its treatment**,** attitudes and beliefs towards taking medicine, and perceptions of medication adherence

Research question:

• What are the factors contributing to poor medication adherence in patients with chronic disease in LMICs?

The sub-questions were:

a) What knowledge do patients have regarding their chronic medication taking?
b) What is the patient's experience with medication adherence?

### 2.2 Information sources and search strategy

We identified the relevant studies by conducting a systematic literature search and included relevant materials on adherence to chronic medication published between 2008 to 2018. Data was sourced from the following databases: PubMed, Google Scholar, EBSCOhost (CINAHL, Health Source, and Medline with full text). Guidelines on policies were searched from different websites, such as government websites and the WHO concerning adherence to chronic medication in LMICs. We used the following keywords to search for eligible literature: chronic patients, adherence, and medication. The Boolean terms were used to separate keywords in the study (AND; OR). The Medical Subject Headings (MESH) were utilized in our search for eligible studies. We included studies in any language. The World Bank countries classification by income level (2018) was used in this study to determine the appropriate categories of the study selected. Refer to Table 2 for search strategy.

**Table 2:** Search strategy from pubmed.

Search number and date	Data base	Retrieved studies	Eligible studies	Search terms
#1. 23/03/2018	PUBMED	2590	70	(("chronic disease"[MeSH Terms] OR ("chronic"[All Fields] AND "disease"[All Fields]) OR "chronic disease"[All Fields]) AND ("adherance"[All Fields] OR "adhere"[All Fields] OR "adhered"[All Fields] OR "adherence"[All Fields] OR "adherences"[All Fields] OR "adherent"[All Fields] OR "adherents"[All Fields] OR "adherer"[All Fields] OR "adherers"[All Fields] OR "adheres"[All Fields] OR "adhering"[All Fields]) AND ("medic"[All Fields] OR "medical"[All Fields] OR "medicalization"[MeSH Terms] OR "medicalization"[All Fields] OR "medicalizations"[All Fields] OR "medicalize"[All Fields] OR "medicalized"[All Fields] OR "medicalizes"[All Fields] OR "medicalizing"[All Fields] OR "medically"[All Fields] OR "medicals"[All Fields] OR "medicated"[All Fields] OR "medication s"[All Fields] OR "medics"[All Fields] OR "pharmaceutical preparations"[MeSH Terms] OR ("pharmaceutical"[All Fields] AND "preparations"[All Fields]) OR "pharmaceutical preparations"[All Fields] OR "medication"[All Fields] OR "medications"[All Fields])) AND ((ffrft[Filter]) AND (fft[Filter]) AND (2008/1/1:2018/3/23[pdat]) AND (data[Filter]))

### 2.3 Selection of sources of evidence

After an intensive search, we exported relevant articles to the Endnote X9 library. Following the import of relevant articles by title relevance, we removed duplicates before the abstract screening stage. We then conducted a selection of abstracts and full articles using eligibility criteria utilizing abstract and full title screening forms developed in Google.

Inclusion criteria:

• Patients diagnosed with chronic diseases such as HIV and AIDS, heart diseases, type II diabetes, hypertension, chronic obstructive pulmonary disease, and chronic kidney diseases
• All patients taking chronic medication
• Studies reporting on any chronic disease published from 2008 to 2018
• Studies focusing on LMIC populations

Exclusion criteria.

• Evidence of disease other than a chronic disease; any kind of communicable and noncommunicable diseases that respond to treatment and do not persist for more than three months
• Studies of patients not taking chronic medications
• Studies published before 2008 and after 2018
• Studies focusing on populations in high-income countries
• Studies focusing on high and low-income populations simultaneously
• Literature reviews and general reports

The selections for the abstracts and full articles were conducted by two independent reviewers (M.E. and T.S.). The third screener (PM) was involved in resolving any discrepancies. Subsequently, we assessed disagreement using Cohen's Kappa coefficient (κ) statistic on Stata 13.0SE (StataCorp College Station, TX, USA) Refer to Additional file 1 (Table 5 and Figure 2).

### 2.4 Data charting process

We developed a data extraction sheet and piloted it before use. In order to demonstrate the important aspects of the study, extractions came from the following domains: author and year; study title; study setting; adherence; knowledge of the disease; attitudes towards the medication; beliefs about disease and medication; perceptions of medication adherence; the aim of the study; study design; outcomes; relevant findings and significant findings. Other information included in the data extraction was the age of the participants, the type of disease, and their gender. The data charting form was updated continually.

### 2.5 Data items

We employed the following data variables:

**Adherence**- Refers the extent to which a patient acts in accordance with the prescribed interval and dosage of medication, and the attitude that the patient has in relation to the agreed recommendation from the health practitioner [15, 16].

**Attitude** – The way in which the patients view and evaluate medication adherence [17].

**Anxiety** – Refers to the strong desire to hold back the unpredictable feeling towards medication adherence [18].

**Beliefs** - The mental ability of chronic patients and acceptance of the facts or actuality of adhering to the prescriptions [19].

**Chronic Diseases** - Refers to illnesses which occur for a period of more than six months without going away, irrespective of any medication being taken, and which may lead to death or disability [20, 21].

**Chronic medication-** - Refers to medicines which are taken by a patient for a period of more than six months without stopping, defaulting may lead to drug resistance, death or disability [20, 21].

**Chronic patient** – An individual taking or registered to get medical treatment for a period of more than three months [22].

**Health care system** – The organisation of people, institution and resources to provide treatment to the patients [23].

**Health literacy** – The patient's knowledge, motivation, competencies to attend and appraise and to communicate health messages and to take control of everyday life [24].

**Knowledge of disease** – The beliefs and accumulation of information that a patient has about chronic diseases [25].

**Low-and Mid-Income Countries-** Refers to the World Bank classification for world countries relying on the Gross National Income (GNI) per capita ranging from $1,036 dollars or less to $ 4,045 [26].

**Medications** – It is regarded as the chemicals or substances used for treatment [27].

**Medication adherence** – The patient's ability to adhere to their prescription on a daily basis and whether they continue with their prescription [28].

**Poor medication adherence** - The way in which the patient exhibits ineffective management of medication, in terms of taking a prescription, following proper diets and executing a lifestyle change [28]**.**

**Quality of life** – The way in which a patient evaluates the goodness of different aspects of their life [29].

**Self-efficacy** – The personal belief about the ability of the self to plan and do actions to achieve and fulfil medication adherence requirements [29].

### 2.6 Critical appraisal of individual sources of evidence

We used the 2018 version of the mixed method appraisal tool (MMAT) to determine and evaluate the risk of bias for each study included [30]. The MMAT consists of five appraising studies sections: Section one focuses on the qualitative component. Sections two, three, and four focus on the quantitative component and section five on the mixed method component (refer to Additional file 2, Table 6). The method used was adequate; therefore, the study design, study selection, data collection, data analysis, presentation of findings, and author's discussion were useful to determine the conclusion of the study. Inputs and scrutiny from all mentioned aspects determined the quality of the article produced. Independent reviewers, DG and DK, contributed by conducting the quality assessment. The percentage obtained in total from each included study was calculated and interpreted as follows: < 50% – low quality, 50% to <75% – average quality, and 75%–100% – high quality.

### 2.7 Synthesis of results

This phase was carried out in three distinct steps. First, the data extracted was based on the characteristics of the studies, i.e., author and year of publication, study title and setting, income level, and type of medication. Second, results were summarized based on the research question and specific objectives. Thus, the information on factors influencing poor medication adherence among chronic patients were summarised using thematic content analysis (e.g., knowledge, attitude). Lastly, the clinical and research implications were described.

## 3 Results

### 3.1 Selection of sources of evidence

**Figure 1:**
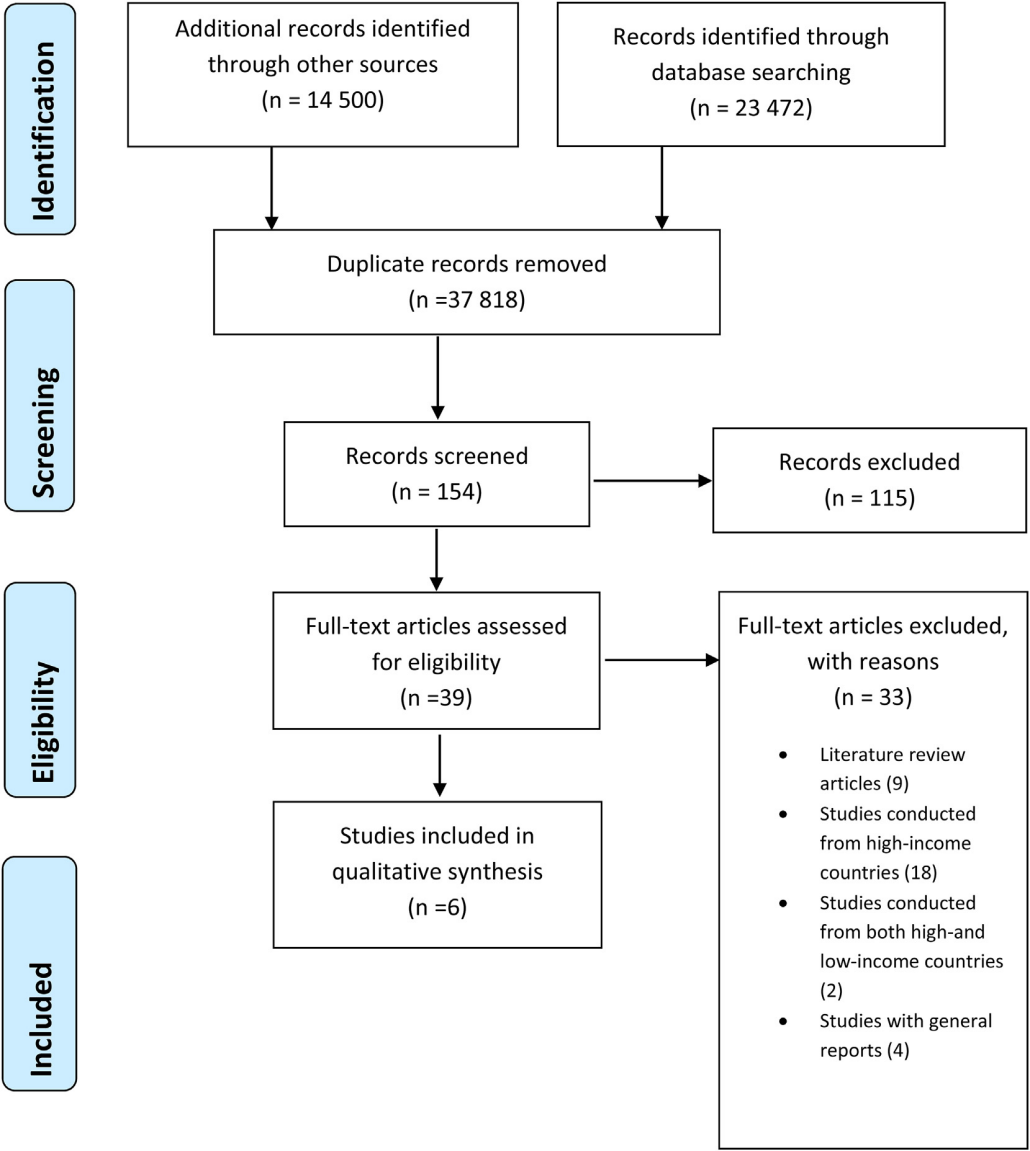
Literature search and selection of eligible studies.

A total number of 37 972 articles were initially identified after title screening. We removed duplicates, which resulted in the exclusion of 37 818 articles.) Studies were searched from the databases using similar keywords hence resulting in similar records producing duplicates. A total number of 154 abstracts were then screened. Of these, 39 were selected for full article screening. After full articles screening, we excluded 33 articles, and the remaining Six (6) met the inclusion criteria and were included for data extraction. Refer to Figure 1. The reasons for the exclusion of the 33 articles following full article screening were as follows: nine (9) studies were literature reviews [22, 31, 32, 33, 34, 35, 36, 37, 38]; eighteen (18) studies [39, 40, 41, 42, 43, 44, 45, 46, 47, 48, 49, 50, 51, 52, 53, 54, 55, 56] were conducted in high-income countries; two (2) studies [57, 58] presented evidence from both low- and high-income countries and four (4) studies were general reports [59, 60, 61, 62].

**Figure 2:**
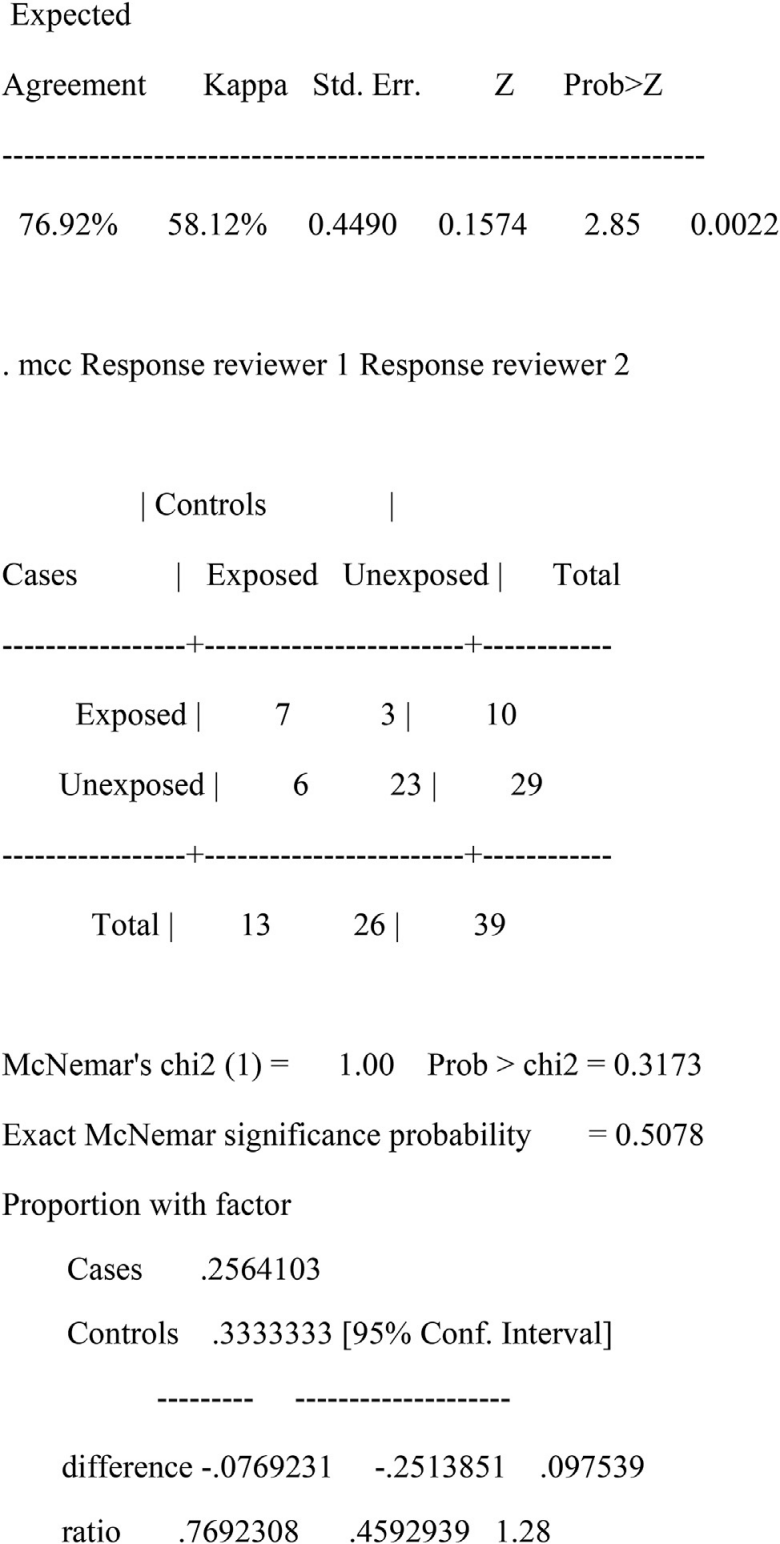
Kappa Response reviewer 1 Response reviewer 2.

### 3.2 Characteristics of sources of evidence

The characteristics of the studies included are presented in Table 3 and Table 4. All six studies included presented evidence on chronic disease and medication adherence. All six studies were conducted in LMICs. The studies were published between 2008 and 2018. The setting for studies were as follows: Jordan [63], Guatemala [64], Ethiopia [65], Indonesia [66], India [29] and Palestine [67]. Two study designs were utilized for studies, including four (4) cross-sectional designs [63, 64, 65, 67] and two (2) randomized controlled trials [29, 66].

**Table 3:** Socio-demographic characteristics of included studies.

Author and Year	Study title and setting	Study population, age (year)	Income level	Type of medication
Awwad *et al.*,	The influence of patients' knowledge	Male and female patients	Middle	Long-term therapy
2015	on adherence to their chronic medications:	taking chronic medication,	income	for one or more
[63]	a cross-sectional study in Jordan.	18 + years		chronic illnesses
Ramay *et al.*, 2017	Factors associated to acceptable treatment	Male and female pediatric	Low-middle	Peritoneal dialysis,
[64]	adherence among children with	patients with Stage Five	income	Haemodialysis and
	chronic kidney disease in Guatemala.	kidney disease		Transplant medication
Gebru et al., 2013	Adherence to Diabetes Self-Management	Male and female patients with	Low income	Oral Hypoglycaemic
[65]	Practices among Type II Diabetic Patients in Ethiopia:	type II diabetes,		Agent and
	A cross-sectional study	30–85 years		insulin therapy
Wati *et al.*, 2017	Hypnodialysis for anxiety relief and adherence	Male and female patients with	Low-middle	Hypnotherapy
	to medication, kidney diet, and fluid intake in	chronic kidney disease,	income	
[66]	patients with chronic kidney disease, Indonesia	All ages		
Ramanath *et al.*, 2012	A study on impact of clinical pharmacist interventions	Male and female rural	Low-middle	Antihypertensive
	on medication adherence and quality of life	hypertensive patients,	income	Medication
[29]	in rural hypertensive patients, India.	18 + years		
Waleed et al., 2014	Influence of patients' disease knowledge and beliefs	Male and female patients	Low-middle	Anti-diabetic Insulin
[67]	about medicines on medication adherence:	with type II diabetes,	income	Therapy
	findings from a cross sectional survey among	18 + years		
	patients with Type II diabetes mellitus in Palestine.

**Table 4:** Aims and outcomes of included studies.

Author and year	Aims and objective	Type of intervention	Outcome: Evidence of knowledge, attitudes, and beliefs
Awwad *et al.*, 2015 [63]	To examine the relationship between knowledge and adherence	Educational interventions targeted	**Knowledge:** Moderately knowledgeable,
	of patients receiving long-term therapy for one or more chronic	at increasing awareness of	with majority lacking aspects related to their medications:
	illnesses in Jordan.	adherence to medication and	side effects, behaviour towards missing doses
		effective ways to remind patients	and when to take the medication
		about their medications.	Significant correlation between patients' knowledge
			and adherence to medications (r = 0.357, p∖0.001).
			Younger age, higher education levels, high income,
			fewer medications and diseases were significant predictors
			of higher knowledge levels. Knowledgeable patients were found
			to be twice as likely to have moderate-high adherence as their less
			knowledgeable counterparts. Higher-income, education was
			associated with higher adherence scores.
			**Attitude:** Forgetfulness and aversion toward medications
			were the most common barriers to medication adherence.
			**Belief:** No evidence on belief
Ramay *et al.*, 2017	To identify the predisposing factors, enabling factors	Educational interventions on the	**Knowledge**: Positive adherence association between
[64]	and need factors related to medication adherence	significance of medication adherence,	mother's educational level and higher monthly income
		Public policy strategies aimed at	
		improving access to comprehensive	**No evidence on attitude and belief**
		treatment regimens	
Gebru *et al.*, 2013	Assess adherence to diabetes self-management practices and	Exercise and change of diet	**Knowledge:** association between age, marital status,
[65]	its associated factors among Type II diabetic patients in Ethiopia.		level of education, monthly income,
			diabetes complication and adherence.
			**Attitude:** All respondents adhered to the recommended
			diabetic foot care practices.
			**Belief:** No evidence on belief
Wati *et al.*, 2017	To examine the effectiveness of hypnodialysis on anxiety levels	Hypnodialysis	**Knowledge**: significant effect of hypnodialysis in reducing
[66]	and adherence to medication, kidney diet and fluid intake		anxiety levels and improving medication
	in patients with chronic kidney disease.		adherence and adherence to kidney diet and
			fluid intake in patients with chronic kidney
			disease with p-value 0.000 (<0.05).
			**Attitude:** Hypnodialysis is beneficial for
			patient's chronic kidney disease who undergo
			Haemodialysis with positive suggestions
			**Belief:** No evidence
Ramanath *et al.*, 2012	To know the impact of clinical pharmacist interventions	Patient counseling,	**Knowledge:** Clinical pharmacist interventions
[29]	on medication adherence and quality of life.	patient information leaflets (PILS.)	among rural population has a strong positive impact
		frequent telephoning reminders.	in creating awareness about the disease and adherence
			**Attitude:** The mental strength/stamina is directly proportional to
			physical activity
			**Belief:** No evidence
Waleed *et al.*, 2014	To assess medication adherence and its potential association	A brief screening was conducted.	**Knowledge:** Disease-related knowledge was significantly
[67]	with beliefs and diabetes-related knowledge		associated with non-adherence: [O.R = 0.87, 95% CI of 0.78–0.97].
	in patients with type II DM.		Diabetic patients with a high knowledge score
			and those with strong beliefs in the necessity of
			anti-diabetic medications were less likely to be non-adherent
			[O.R = 0.93, 95% of 0.88–0.99].
			**Attitude:** forgetfulness, concerns about adverse consequences
			of the chronic use of anti-diabetic medications
			were significantly associated with nonadherence
			
			**Beliefs:** Medicines in general
			are essentially harmful, and this is significantly
			associated with nonadherence
			[O.R = 0.87, 95% CI of 0.78–0.97].

**Table 5:** Full article screening-degree of agreement.

No	Author and date	Response: reviewer 1	Response: reviewer 2
1	Adliah, MA *et al* 2012	No	Yes
2	Awwad, O. *et al* 2015	Yes	Yes
3	Badawy, S. M. *et al* 2017	No	No
4	Brown, Marie T *et al* 2011	No	Yes
5	Chan, W.; *et al* 2017	No	No
6	Chopra, A. S. *et al* 2018	No	No
7	Fischer, W. *et al* 2018	No	No
8	Kalayou Kidanu Berhe et al 2013	Yes	Yes
9	Ha Dinh, T. T*. et al* 2016	Yes	No
10	Hamine, *Saee et al* 2015	No	Yes
11	Haynes, *et al* 2008	No	No
12	Hirt, M. N. *et al* 2016	No	No
13	Jacobs, A *et al* 2014	No	No
14	Kearns, *et al* 2017	No	Yes
15	Krauskopf *et al* 2015	Yes	No
16	Kreps, G. L. *et al* 2011	No	No
17	Laufs, U.*et al* 2011	No	No
18	Mann *et al* 2009	No	No
19	Manteuffel *et al* 2014	No	Yes
20	Matthew *et al* 2018	Yes	Yes
21	Mishra *et al* 2011	No	No
22	N. Aujla, *et al* 2016	No	No
23	Pages-Puigdemont, *et al* 2016	No	No
24	Piette, J. D. *et al* 2011	No	No
25	Qian *et al* 2014	No	No
26	Ramanath, K *et al* 2012	Yes	Yes
27	Ramay, B. M *et al* 2017	Yes	Yes
28	Schreibman *et al* 2016	Yes	No
29	Sontakke *et al* 2015	No	No
30	Their *et al* 2008	No	No
31	Tourkmani *et al* 2012	No	No
32	Traynor, K. *et al* 2012	No	No
33	Umeukeje, *et al* 2016	No	No
34	UNDP 2013	No	Yes
35	Waleed M 2014	Yes	Yes
36	Wati *et al* 2017	Yes	Yes
37	Zaugg, V. 2018	No	No
38	Zomahoun *et al* 2017	No	No
39	Zullig, *et al*; 2013	No	No

**Table 6:** MMAT Quality assessment.

Study Type	Methodological Criteria
Screening questions (All study types)	➢ Are there clear research questions/objectives? ➢ Do the collected data address the research questions/objectives?
Quantitative	➢ Is the sampling strategy relevant to address the quantitative research question? ➢ Is the sample representative of the population under study? ➢ Are measurements appropriate (clear origin, or validity known, or standard instrument)? ➢ Is there an acceptable response rate (60% or above)?
Qualitative	➢ Are there sources of qualitative data relevant to address the research question or objective? ➢ Is the process for analyzing qualitative data relevant to address the research question or objective? ➢ Is appropriate consideration given to how findings relate to the context in which the data were collected? ➢ Is appropriate consideration given to how findings relate to researchers' influence?
Mixed methods	➢ Is the mixed methods research design relevant to address the qualitative and quantitative research question (or objectives)? ➢ Is the integration of qualitative and quantitative data (or results) relevant to address research question (objectives)? ➢ Is appropriate consideration given to the limitations associated with this integration, e.g. the divergence of qualitative and quantitative data (or results) in a triangulation design?

### 3.3 Critical appraisal within sources of evidence

The quality of evidence from included studies indicates that five studies scored 100%, which is regarded as the highest score [63, 64, 65, 66, 67]. The remaining study scored between 50–75% [29], indicating a moderate to low risk bias.

### 3.4 Results of individual sources of evidence

Through thematic analysis, the following factors were found to influence poor medication adherence: knowledge of disease and treatment, attitude towards adherence to chronic medication, and beliefs about taking medicines. We included two other emerging themes: quality of life and anxiety.

#### 3.4.1 Knowledge of the disease and treatment

All six included studies reported on lack of knowledge as one of the factors influencing poor adherence among patients taking chronic medication [29, 63, 64, 65, 66, 67]. All six studies reported a lack of knowledge as a barrier to medication adherence in both male and female patients. Evidence showed a correlation between patients' knowledge about the disease, treatment, and adherence [63]. Study findings from Jordan(2015) revealed that a patient's educational level contributes to medication adherence [63]. It was demonstrated that a higher level of education was significantly associated with medication adherence [65]. A study conducted in India shows poor medication adherence after the patients were introduced to clinical pharmacist interventions, consequently improving their knowledge [66]. A study aimed at assessing chronic medication adherence and its potential association with knowledge in Palestine demonstrated that chronic patients' knowledge of their disease influences their adherence to medication [63]. Our findings indicate a gap in knowledge surrounding medication adherence in patients taking chronic medication in low- and middle-income countries [65].

#### 3.4.2 Attitude towards adherence to chronic medication

Five studies reported patients' attitudes towards adherence to chronic medication [29, 63, 64, 65, 66, 67]. Awwad *et al.* (2015) found that nonadherence was associated with patients forgetting to take their medications and the negativity toward taking chronic medication [63]. A study conducted in Ethiopia aimed at assessing adherence to diabetes self-management practice and its associated factors among type II diabetes patients has shown that respondents adhered to the recommended diabetic foot care practice [65]. It was evident that patients in this study had sufficient self-care practice to manage and control their chronic diseases and reduce more challenging health conditions [65]. A study from Indonesia aimed to determine the effectiveness of hypnotherapy on anxiety and adherence to medication, kidney diet, and fluid intake in patients with chronic disease indicated that hypnotherapy was effective in improving adherence to chronic medication [66]. Furthermore, a study from India aimed at investigating the impact of clinical pharmacist interventions on medication adherence and quality of life was found to be directly proportional to physical activity [29]. Therefore, paying more attention to mental strength improved the management of patients on treatment [29].

A study from Palestine aimed at assessing anti-diabetic medication adherence and its potential association with beliefs and diabetes-related knowledge among patients with type II DM attending primary health care showed poor adherence to medication [67]. Forgetfulness and concerns about the adverse consequences of anti-diabetic medications' chronic use were significantly associated with nonadherence [67]. However, some patients stopped taking their medication when they felt like their diabetic symptoms improved [67]. Our study findings indicate a gap in the research regarding interventions targeted at reminding patients of the significance of adhering to medication and having a positive attitude towards medication adherence [66].

#### 3.4.3 Beliefs about taking medicines

One (1) study reported on beliefs about taking medicine. This study was conducted among Palestinian adults with type II DM [67]. The study aimed to assess medication adherence and its potential association with beliefs and diabetes-related knowledge amongst patients with type II DM attending a PHC clinic [67]. The study showed that diabetic patients with strong beliefs about chronic disease were less likely to be non-adherent OR = 0.87, 95% CI of 0.78–0.97].

and [O.R = 0.93, 95% of 0.88–0.99] respectively) [67]. Nonetheless, evidence showed that most chronic patients strongly believed that chronic medications were necessary for their present and future health [67]. Further research on chronic medication adherence and beliefs is therefore recommended [67].

#### 3.4.4 Quality of life

Findings on quality of life in relation to medication adherence were reported from one (1) study [29]. The study was conducted in rural India and included patients with hypertension. The aim of the study was to determine the impact of pharmacist interventions on medication adherence and quality of life (QOL). Findings indicate that pharmacist interventions through patient counseling, patient information leaflets (PILs), and frequent telephonic reminding can help improve medication adherence by improving their knowledge about adherence as well as their QOL. There is limited evidence of pharmacist interventions impacting QOL. More research should be directed towards other chronic disease management other than hypertension to avoid reoccurrence of disease, poor prognosis and minimize hospital admissions [29].

#### 3.4.5 Anxiety

One (1) study reported anxiety related to adherence to medication [66]. The study was conducted in Indonesia and included male and female patients with chronic kidney disease [66]. The study examined the effectiveness of hypnoanalysis on anxiety levels and adherence to medication, kidney diet, and drinking sufficient water in participants with chronic kidney disease [66]. The study mainly concentrated on hypnotherapy as an intervention to influence patients to adhere to kidney medication [66]. It was evident in this study that the patients changed their habits and behaviour regarding taking their prescribed medication [66]. There was no clear research gap in the literature regarding hypnoanalysis improving medication adherence in patient**s** with chronic disease.

### 3.5 Synthesis of results

We provided a narrative account of the evidence revealed from the studies included. The data was analyzed using thematic content analysis. The review team analyzed the implications of data findings to check how they related to the research aims and objectives.

## 4 Discussion

This systematic scoping review mapped evidence of factors influencing poor medication adherence amongst chronic patients in LMICs. Until now, to our knowledge, this is the first scoping review that focuses on mapping evidence on factors influencing medication adherence amongst chronic patients in LMICs. Various factors were identified as influencing poor chronic medication adherence in LMICs. The factors include knowledge of the disease and treatment, attitude towards adherence to chronic medication, beliefs about taking medication, and quality of life.

Studies on chronic medication adherence factors have been conducted extensively in high and middle-income countries (HICs); however, less information is evident on LMICs [29, 66, 67]. Nonetheless, our study findings on knowledge and attitude [63, 64, 67, 68, 69] correlate with other studies conducted in LMICs [70, 71, 72]. Findings from one of the studies conducted in LMICs reveal that forgetfulness, knowledge about diabetes and its medications, and side effects are two of the factors associated with medication adherence among patients with diabetes in the Middle East and North Africa region [70]. In agreement with results from the Middle East and North African region is a study aimed at improving diabetes in LMICs [72]. The study argues that improving diabetes through a health dialogue intervention increases awareness [72]. Thus, this shows how educational interventions can significantly change the outcome of chronic medication adherence, as suggested by Abbasi et al. (2018) [71]. Magadza., Radloff., and Srinivas (2009) from South Africa confirm that educational interventions increased the participants' levels of knowledge about hypertension and thus positively influenced their beliefs about chronic medication [73].

Furthermore, Konstantinou P et al. (2020) states that interventions such as digitally delivered interventions, including components such as medication and condition education, motivational interviewing (MI), and reinforcement and motivational messages, led to improvements in medication adherenc [74]. We support the strategies mentioned by Konstatinou P et al. (2020); hence the strategies could be considered to fill the gap of forgetfulness, aversion to medication, and negative attitude about chronic medication in relation to the results on attitude obtained in our study [63, 67]. Ahmadi et al. (2019) also support the intervention of reminder messages. Even though Ahmadi researched chronic hyperlipidemia, this can still apply to our study findings. A scoping review that was conducted on medication adherence across chronic conditions including asthma, cancer, diabetes, epilepsy, HIV/AIDS, and hypertension reports that the most commonly reported barriers to medication adherence across conditions were but not restricted to low education, side effects, patient beliefs/perceptions, and poor patient-provider communication. These factors are similar to those identified in our study [63, 64, 67, 68, 69]. Interventions such as SMS reminders could work in relation to responding to these barriers.

Different from our study findings is a study conducted in Iran where patients had a different belief that medication was harmful and that the drugs were over-prescribed by doctors [75]. The study recommends that patients be encouraged to express their views on medications to improve patients beliefs about medication [75]. Positive belief in the efficacy of antihypertensive medication is associated with chronic medication adherence [73]. The perception of patients taking chronic medication has been studied intensively, but less is evident about their impact on medication adherence [76].

Even though evidence on quality of life is limited in our study, studies conducted in South Africa, Bangladesh, Pakistan, Sri Lanka, and South Korea have demonstrated that quality of life is associated with medication adherence, especially hypertension [77, 78, 79]. These studies further demonstrated that educational interventions, organizational interventions aimed at delivery care, and sms reminder systems could effectively manage chronic medication adherence [77, 78, 79]. Adequate knowledge is important as it improves the quality of life [29]. It is evident that poor medication adherence among chronic patients in LMICs is existent [29, 63, 64, 68, 69, 80].

### 4.1 Recommendations for future research

As medication adherence is one of the major health problems in LMICs, specific questions need to be addressed: Are there any resources to assist the LMICs in achieving more effective health outcomes? Are there government-funded strategies or research-intensive studies focused on factors influencing medication adherence? For future research, we also recommend researchers to conduct systematic reviews and meta-analyses in LMIC settings on specific chronic diseases such as hypertension in order to come up with knowledge on the effectiveness of interventions toward medication adherence.

### 4.2 Implications for the practice

Evidence from this scoping review indicates several challenges that chronic medication patients encounter daily. Some of the measurement tools for medication adherence were translated from English to Arabic, and therefore, validity may be questionable [67]. In most LMICs, there is a need to explore interventions that inform chronic medication patients about implications due to nonadherence to influence chronic medication behavior such as forgetfulness and reluctance in patients taking chronic medication [63]. Understanding and considering the issue of literacy in LMICs, policies enhancing communication challenges that patients are facing need to be in place. To maximize the success of medication adherence and reduce the tendency of drug resistance, follow-up programs should be encouraged for patients who miss or skip their subsequent visits [81].

### 4.3 Strengths of the study

An intensive and exhaustive search was conducted to -identify the relevant studies to answer our research question. The Boolean terms and MeSH were used during the database search to increase the chances of finding eligible studies. Screening and data extraction tools were piloted to increase the reliability of the study. Reliability was supported by measuring the degree of agreement between different study results to rule out any bias. We also assessed the quality of the studies included by utilizing the MMAT.

### 4.4 Limitations

The World Bank index is continuously updated, and the classification of countries keeps on changing. For example, it could be found that a country, which was classified as being in a low-income to a middle-income country in 2018, would be in the category of a high-income country the following year. This limitation indicates that it is vital to constantly research these countries that changed categories. Further research needs to be conducted about medication adherence in LMICs.

## 5 Conclusions

Our scoping review provides evidence that poor medication adherence in LMICs is influenced by a lack of knowledge, negative attitudes, and negative beliefs, leading to poor quality of life. Even though the factors mentioned earlier were identified, studies on beliefs and quality of life were limited in LMICs. We call upon studies focusing on beliefs or perceptions and quality of life to understand these factors in the current setting better.

## Declarations

### Author contribution statement

Gloria Dunisani Chauke and Tivani Mashamba-Thompson: Conceived and designed the experiments; Performed the experiments; Analyzed and interpreted the data; Contributed reagents, materials, analysis tools or data; Wrote the paper.

Olivia Nakwafila: Performed the experiments; Analyzed and interpreted the data; Contributed reagents, materials, analysis tools or data; Wrote the paper.

Buyisile Chibi: Performed the experiments; Analysed and interpreted the data.

Benn Sartorius: Analyzed and interpreted the data.

### Funding statement

This work was supported by the 10.13039/501100004695University of KwaZulu-Natal [College of Health Sciences Research Scholarship].

### Data availability statement

Data included in article/supp. material/referenced in article.

### Declaration of interests statement

The authors declare no conflict of interest.

### Additional information

No additional information is available for this paper.
